# Exploring Patient Mealtime Experience in an Acute Care Setting Using the Modified Austin Health Patient Mealtime Experience Tool

**DOI:** 10.1111/jhn.70068

**Published:** 2025-05-28

**Authors:** Laura Lam, Helen Ussher, Gina Trakman, Amy Daglas, Ella Hamilton, Lauren Ballantyne, Virginia Fox, Kate Furness

**Affiliations:** ^1^ Department Sport, Exercise and Nutrition Sciences, School of Allied Health, Human Services and Sport La Trobe University Bundoora Victoria Australia; ^2^ Nutrition and Dietetics Department Bendigo Health Victoria Australia

**Keywords:** dietetics, food services, hospital, malnutrition, mealtime care, patient experience

## Abstract

**Aims:**

Malnutrition is prevalent in Australian hospitals, affecting 30%–40% of inpatients. Enhancing patient mealtime experience is a recognised strategy to support improved dietary intake and clinical outcomes. Yet, there is little published data on mealtime experience in acute hospital settings in Australia. This study aims to capture patient mealtime experience in an acute care setting at a regional Australian hospital, using a modified version of the Austin Health Patient Mealtime Experience Tool.

**Methods:**

A cross‐sectional study was undertaken across six acute care wards at Bendigo Health between July and September 2024. Patient mealtime experience was explored through interviewer‐administered surveys, including 32 Likert scaling items and 6 open‐ended responses. Descriptive statistics were used to analyse quantitative data, whilst deductive thematic analysis was applied to qualitative data to describe mealtime experience.

**Results:**

Eighty‐one patients participated in the study. Patients were most dissatisfied with food quality, particularly sensory characteristics and variety, in both the quantitative and qualitative results. Patients were most frequently satisfied with staff interactions (90% ‘always’ or ‘often’ positive), although the qualitative results highlighted insufficient mealtime care. The physical environment was generally highly rated, with a majority of patients (> 70%) reporting that noise, visitors, room surroundings and smells and odours ‘rarely’ or ‘never’ impacted food intake. The food ordering system was rated favourably, with 89% of participants rating meal timing as ‘always’ or ‘often’ positive and 73% rating meal accuracy as ‘always’ or ‘often’ satisfactory. However, qualitative results revealed usability issues related to the electronic meal ordering system. Finally, qualitative responses identified nutrition impact symptoms as a barrier to mealtime experience and intake.

**Conclusion:**

Food quality, sufficient mealtime care, management of nutrition impact symptoms and improving usability of electronic ordering systems are areas highlighted for improvement in mealtime experience. Addressing these factors through targeted quality improvement initiatives can enhance mealtime satisfaction and support nutritional intake. Integrating patient perspectives into service planning is essential for fostering patient‐centred hospital foodservices and improving patient outcomes.

## Summary

- In this sample of acute care patients, food quality, insufficient mealtime care, usability issues with the electronic meal ordering system (EMO) and nutrition impact symptoms were key areas negatively impacting patient mealtime experience (MTE).
- These results can be used to inform quality improvement initiatives to improve MTE and support nutritional intake in acute care patients.
- Integrating patient perspectives into service planning is essential for a more patient‐centred hospital foodservice experience, highlighting the need for ongoing MTE monitoring and evaluation.

## 1 Background

The European Society for Enteral and Parenteral Nutrition (ESPEN) defines malnutrition as ‘a state resulting from a lack of uptake or intake of nutrition leading to altered body composition (decreased fat‐free mass and body cell mass) leading to diminished physical and mental function and impaired outcome from disease’ [1]. In Australia, malnutrition affects 30%–40% of hospital inpatients [2, 3, 4] and is associated with adverse outcomes, including muscle wasting, frailty, impaired wound healing, increased rates of infection and increased risk of developing pressure injuries [2, 3, 5, 6, 7]. From a broader perspective, malnutrition is linked to increased morbidity and mortality, lengthened hospital stays, risk of readmission and ultimately, increased healthcare expenditure [2, 3, 5, 7]. Inadequate dietary intake, driven by factors such as anorexia, impaired nutrient absorption and/or utilisation, increased nutrition requirements and procedure‐related fasting are key contributors to malnutrition [8, 9, 10, 11]. Despite advances in nutrition support such as food fortification and oral nutritional supplementation, malnutrition continues to be prevalent in hospitals worldwide [11]. A large proportion of inpatients are malnourished on admission, with the condition being exacerbated during their hospital stay [11]. Well‐nourished patients are at risk of developing malnutrition due to factors in the hospital environment [11].

Recently, there has been an increased focus on identifying and addressing modifiable components contributing to poor dietary intake in healthcare settings [11]. One such component is patient mealtime experience (MTE) − a critical component of overall patient experience [11, 12, 13, 14]. Patient experience can be described as ‘what the process of receiving care feels like for the patient’ [15] and is considered a key element of quality care, alongside providing clinical excellence and safer care. Higher levels of overall patient experience have been increasingly linked to improved health outcomes [16]. As an indivisible part of the broader patient experience, MTE is a complex, multidimensional construct encompassing patient satisfaction with food quality; food access and choice; the mealtime environment; the food ordering system; service reliability; and interactions with hospital staff [8, 12, 14, 17, 18, 19].

At the same time, there is growing attention to the relationship between patient‐centred care and improved health outcomes [20, 21]. This paradigm shift positions patients not only as recipients of care but also as active participants [20]. The Australian National Safety and Quality Health Services (NSQHS) emphasises the significance of patient preference, experience and involvement in their care and in quality improvement [22]. Action 1.13 of the NSQHS Standards mandates that healthcare services implement regular feedback mechanisms from patients regarding their care experiences to enhance safety and quality systems [23]. As health services are under increasing pressure to evaluate and report patient satisfaction outcomes, the measurement of MTE becomes critical.

In Australia, the Acute Care Hospital Foodservice Patient Satisfaction Questionnaire (ACHFPSQ) [24] is a recognised and recommended tool for the measurement of food service satisfaction. Although this tool is valid and reliable [24, 25], it does not fully capture critical elements of MTE including food ordering systems, mealtime assistance and other factors that impact adequate intake [12, 24]. Additionally, the ACHFPSQ neglects to consider patients' subjective experiences with food services and mealtime care [12]. As patient‐centred care becomes increasingly prioritised in healthcare delivery, there remains a need for questionnaires to capture qualitative feedback from patients to enhance data sensitivity and to develop actionable strategies for improving food services [12, 25].

In 2023, the Austin Health Patient Mealtime Experience Tool (AHPMET) was developed to capture the breadth of factors that impact MTE whilst incorporating open‐ended questions to gather qualitative feedback from patients [12]. This holistic approach allows the AHPMET to enrich the data available for strategic planning and quality improvement [12]. The triangulation of quantitative data with qualitative feedback ultimately provides a more comprehensive understanding of the patient experience, supporting a more responsive and person‐centred approach to care [16]. The AHPMET tool has recently undergone formal validation, with findings currently pending publication.

Whilst the AHPMET has previously been pilot tested in an acute population, data collection in a previous study was limited to a subacute and rehabilitation setting in a metropolitan hospital due to COVID‐19 restrictions [12]. There remains a need to extend this evaluation in an acute care setting, where MTE may be influenced by the unique challenges and demands experienced by this patient population. The aim of this study is to capture patient mealtime experience in an acute care setting at a regional Australian hospital, using a modified version of the AHPMET tool.

## 2 Methods

### 2.1 Study Design, Setting and Sampling

This cross‐sectional study was undertaken at Bendigo Health, a 724‐bed tertiary regional hospital (Bendigo, Australia). Bendigo Health is a publicly funded health service serving approximately 334,000 people across the Loddon Mallee region in northern and central Victoria [26].

Bendigo Health has a unique electronic menu ordering system (EMO) known as the Patient Entertainment System (PES) with the capacity for patients to order meals. The EMO displays the menu using English text, with an appearance similar to a traditional paper menu. Patients are able to view ingredients included in the menu item, accessible via a small icon next to the menu item. Additionally, patients are able to see the names of the available menu items and select their choices for the following day. Patients who do not complete a meal order via the EMO are alerted to menu monitors, who then visit the patient to take their meal order. Bendigo Health has a policy in place to support mealtime care through a Mealtime Assistance and Environment Protocol.

This study was conducted across six acute care wards (Intensive Care Unit (ICU); surgical; orthopaedics; respiratory; and two general medicine wards). Wards were selected to capture a range of clinical contexts and patient care needs to increase the generalisability and dependability of the data. Participants were recruited using a cross‐sectional sampling approach. All eligible and consenting patients on the selected wards were invited to participate over a 3‐week period. Patients were approached on each ward on repeated weekdays to maximise recruitment and to account for patient turnover. This study was conducted by three La Trobe University Master of Dietetics students (L.L., A.D. and E.H.) over two phases of an inquiry‐based research placement, with supervision by Bendigo Health (H.U. and L.B.) and La Trobe University staff (K.F. and G.T.).

### 2.2 Survey Instrument

Minor modifications were made to the abovementioned AHPMET instrument to capture Bendigo Health food service processes. This included the removal of a question regarding the use of a dining room, as the Bendigo Health facility does not include an inpatient dining room. Additionally, a question to gather patient feedback on how meals are ordered from the hospital menu was added to explore patient use of the EMO. Two additional questions (6 and 7) were added to capture compliance with Bendigo Health's internal Mealtime Assistance and Environment Protocol. The final tool (Supporting Material S1) consisted of 32 Likert scaling items (1 = never to 5 = always, Not Applicable = 0) and six open‐ended questions grouped into four dimensions of MTE: Food Quality, Environment, Staff Interactions/Assistance and the Food Ordering System. Five closed‐ended questions capture demographic data, including age, hospital diet code, length of hospital stay, country of birth and sex.

### 2.3 Inclusion/Exclusion Criteria

Participants were recruited from July to September 2024. Adult inpatients who received or were eligible to receive meals for at least one full day (breakfast, lunch and dinner) were considered for inclusion in this study. Patients in the following wards were considered for inclusion in this study: Intensive Care Unit (ICU); surgical; orthopaedics; respiratory; and the two general medicine wards. Patients who were not eligible to receive meals (e.g. patients with nil‐by‐mouth status) were excluded from the study. Additionally, patients under 18 years of age, those with an admission diagnosis of mental illness or otherwise identified by nursing staff as unsuitable for interview (i.e. due to significant cognitive impairment, medical symptoms or non‐English speakers) were excluded.

### 2.4 Data Collection

Researchers liaised with Nurse Unit Managers and/or Assistant Nurse Unit Managers to identify suitable participants. Daily patient lists for each ward were reviewed during the data collection period to ensure a systematic and comprehensive approach to identify eligible participants and to account for ward turnover throughout the data collection period. All patients who met the inclusion criteria were approached in person by one of three researchers for participation. To reduce inter‐person variability, a standardised verbal script was employed to provide patients with a description and purpose of the study and information as to voluntary participation (Supporting Material S2). Verbal consent was obtained.

All surveys were interviewer‐administered. All survey questions, including open‐ended items, were asked verbatim as worded in the AMPHET tool to promote procedural rigour in data collection. Open‐ended questions were transcribed verbatim at the time of the interview. Data were digitally compiled in a custom‐designed Excel document and cross‐checked with a second researcher for accuracy. To ensure rigour, the raw hard copy data were cross‐checked with the raw digital entries, and discrepancies were corrected. Categorical data were coded numerically at the time of digital data entry.

### 2.5 Ethics

This study was classified as low risk and met the Bendigo Health Quality Assurance threshold. No formal ethical approval was required. No identifiers or clinical information was obtained from the survey, except for demographic and contextual data, including diet codes and length of hospital stay. Data were stored securely in a Bendigo Health network drive, accessible only by the researchers.

### 2.6 Researcher Positioning

The researchers (L.L., A.D. and E.H.)are student dietitians and had no affiliation with Bendigo Health before the conduct of this study. L.L., A.D. and E.H. previously completed clinical dietetic training at separate healthcare facilities.

### 2.7 Reflexivity

As dietitians in training, the researchers were emotionally invested in advocating for quality nutritional care for the study population [27]. L.L., A.D. and E.H. regularly and continually reflected upon their subjective and instinctive responses during the data collection and data analysis phases and how this may have impacted the construction and interpretation of the findings [27].

### 2.8 Data Analysis

Quantitative data were analysed descriptively using IBM SPSS Statistics (version 29) [28]. Normality testing for continuous variables was conducted using the Kruskal–Wallis test. Age and length of stay were non‐parametric and were presented using median and Interquartile Range (IQR). Categorical data (demographic data, ward, treating team and diet code) are presented as frequencies. Survey Likert scaling data were reported using frequencies and percentages, per established recommendations [29].

Qualitative data were analysed thematically using Excel spreadsheets, employing the Framework Method as a guiding methodology for theme and subtheme development from the survey data to accurately capture patients' perceptions, attitudes and experiences related to MTE [30]. The Framework Method is a structured approach that enables the identification and categorisation of qualitative data which allows for comprehensive comparison and interpretation across cases [30]. The data analysis framework was based on the four dimensions of MTE as set out in the modified AHPMET. Following a deductive approach, two researchers (L.L. and A.D.) independently coded 20% (*n* = 16) of the data set to identify themes and subthemes. Researchers verified and refined themes and subthemes by consensus. These themes and subthemes were then provided with labels and organised into a structured codebook, forming the framework that was applied to all subsequent qualitative data.

## 3 Results

A total of 81 participants completed the modified AHPMET (51% female, 49% male) (Table 1). The median age was 73 years (IQR 24–90) and the median length of hospital stay at the time of data collection was 4 days (IQR 1–75). Fifty‐nine percent (*n* = 48) of participants received a full ward diet. The remainder of participants received therapeutic diets (modified eating plans tailored to meet specific nutritional needs or conditions, such as texture‐modified diets for patients with dysphagia) or a combination of therapeutic diets. Participants most commonly ordered meals through menu monitors (52%, *n* = 42), followed by the PES (27%, *n* = 22) or a combination of the two (10%, *n* = 8).

**Table 1:** Demographic characteristics.

Characteristic	*n* = 81
Age (years), median (IQR)	73 (20)
Gender, *n* (%)	
Female	41 (50.6)
Male	40 (49.4)
Country of birth, *n* (%)	
Australia	70 (86.4)
Other	11 (13.3)
Length of stay (days), median (IQR)	4 (4)
Ward, *n* (%)	
2 F (Intensive Care)	2 (2.5)
4 A (Medical)	15 (18.5)
4B (Orthopaedics)	15 (18.5)
5 A (Medical)	15 (18.5)
5B (Surgical)	21 (25.9)
6 C (Respiratory)	13 (16)
Medical team, *n* (%)	
Endocrinology	1 (1.2)
GEM on Acute	3 (3.7)
General Medicine	33 (40.7)
General Surgery^a^	20 (24.7)
Oncology	3 (3.7)
Orthopaedic Surgery	14 (17.3)
Plastic Surgery	2 (2.5)
Renal	3 (3.7)
Urology	2 (2.5)
Diet Code, *n* (%)	
Diabetic^b^	18 (22.2)
Texture modified^c^	11 (13.6)
Full ward diet	48 (59.3)
High‐protein diet	1 (1.2)
Other	3 (3.6)
Food Ordering Method, *n* (%)	
Menu Monitor	42 (51.9)
PES	22 (27.2)
Menu Monitor and PES	8 (9.9)
Other^d^	6 (13.4)
Not Applicable	2 (2.5)

Abbreviation: PES, patient entertainment system.

^a^ Includes Acute General Surgery.

^b^ Includes diabetic and diabetic/low‐fat/high‐protein diet codes.

^c^ Includes soft bite‐sized and pureed diets.

^d^ Includes meals that are not ordered and/or family/visitors ordering for patients.

## 4 Quantitative Findings

Overall, 55% participants reported that their meals were ‘often’ or ‘always’ enjoyable (item 4h) (Table 2). The poorest‐performing responses in the food quality domain were questions related to the taste and flavour of meals (4g), and the appearance of meals (4f), with 46% and 36% of participants, respectively, rating these as ‘often’ or ‘always’ positive. Conversely, responses most frequently rated as positive in the food quality domain related to the temperature (4e) and serving sizes (4d) of meals, with 70% and 80% of participants rating these as ‘often’ or ‘always’ being suitable and adequate, respectively. Overall satisfaction with food quality (4a) was mixed. Whilst 52% of participants expressed that they were ‘often’ or ‘always’ satisfied with food quality, 42% indicated that they were ‘sometimes’ or ‘rarely’ satisfied.

**Table 2:** Descriptive statistics of likert scaling questions from modified AHPMET tool (*n* = 81).

Item		Not Applicable, *n* (%)	Never, *n* (%)	Rarely, *n* (%)	Sometimes, *n* (%)	Often, *n* (%)	Always, *n* (%)
	**Food Quality**						
4a	How frequently have you been satisfied with the quality of the food you have received at Bendigo Health	1 (1.2)	4 (1.2)	13 (16)	21 (25.9)	27 (33.3)	15 (18.5)
4b	Have the meals offered been appropriate for your beliefs or needs? (e.g. religious, cultural, vegan)?	29 (35.8)	2 (2.5)	1 (1.2)	2 (2.5)	13 (16)	34 (42)
4c	Has there been variety in your meal choices?	2 (2.5)	3 (3.7)	8 (9.9)	9 (11.1)	29 (35.8)	30 (37)
4d	Has the serving size of your meals been adequate?	3 (3.7)	1 (1.2)	4 (4.9)	8 (9.9)	11 (13.6)	54 (66.7)
4e	Have the meals been served at a suitable temperature?	1 (1.2)	0 (0.0)	6 (7.4)	4 (4.9)	13 (16.0)	57 (70.4)
4f	Have the meals looked appetising when they were presented?	2 (2.5)	5 (6.2)	12 (14.8)	26 (32.1)	20 (24.7)	16 (19.8)
4g	Has the taste and flavour of the meals been to your liking?	1 (1.2)	10 (12.3)	12 (14.8)	21 (25.9)	17 (21)	20 (24.7)
4h	Overall, have your meals been enjoyable?	2 (2.5)	10 (12.3)	12 (14.8)	13 (16)	25 (30.9)	19 (23.5)
	**Environment**						
	*Do the following factors affect the amount of food you eat during mealtimes?*						
5a	Noise	6 (7.4)	54 (66.7)	12 (14.8)	6 (7.4)	1 (1.2)	2 (2.5)
5b	Visitors and/or other patients	7 (8.6)	51 (63)	12(14.8)	8 (9.9)	1 (1.2)	2 (2.5)
5c	Room surroundings (e.g. layout of the room, furniture, lighting, ambience)	4 (4.9)	59 (72.8)	9 (11.1)	5 (6.2)	3 (3.7)	1 (1.2)
5d	Interruptions by hospital staff (e.g. wanting to speak to you or give you treatment)	5 (6.2)	35 (43.2)	15 (18.5)	19 (23.5)	6 (7.4)	1 (1.2)
5e	Smells and odours	4 (4.9)	54 (66.7)	4 (4.9)	16 (19.8)	3 (3.7)	0 (0.0)
6	Is your meal tray placed where you can easily reach your food?	3 (3.7)	1 (1.2)	0 (0.0)	5 (6.2)	10 (12.3)	62 (75.5)
7	Is your tray table cleared of clutter (medical products, specimen samples, urinals) when your meal is served?	5 (6.2)	2 (2.5)	9 (11.1)	13 (16)	11 (13.6)	41 (50.6)
	*Over the past 2 days have the following aspects affect the amount of food you eat during mealtimes?*						
8a	Loss of appetite	6 (7.4)	32 (39.5)	8 (9.9)	13 (16.0)	14 (17.3)	8 (9.9)
8b	Nausea and/or vomiting	6 (7.4)	44 (54.3)	7 (8.6)	13 (16.0)	8 (9.9)	3 (3.7)
8c	Pain	5 (6.2)	39 (48.1)	8 (9.9)	15 (18.5)	8 (9.9)	6 (7.4)
8d	Tiredness	4 (4.9)	38 (46.9)	11 (13.6)	16 (19.8)	10 (12.3)	2 (2.5)
8e	Difficulty chewing or swallowing	6 (7.4)	52 (64.2)	7 (8.6)	7 (8.6)	4 (4.9)	5 (6.2)
8f	Position (e.g. your posture, ease of access to food tray)	5 (6.2)	44 (54.3)	11 (13.6)	14 (17.3)	6 (7.4)	1 (1.2)
	**Staff interactions/Assistance**						
9a	Does the meal tray (including cutlery, serviettes, packaging etc.) have everything you need?	1 (1.2)	0 (0.0)	2 (2.5)	8 (9.9)	10 (12.3)	60 (74.1)
9b	Is assistance available if you need help opening the packaging on the meal tray?	20 (24.7)	0 (0.0)	2 (2.5)	8 (9.9)	5 (6.2)	46 (56.8)
9c	When you need help, are staff there to provide assistance at your mealtimes?	21 (25.9)	0 (0.0)	1 (1.2)	7 (8.6)	9 (11.1)	43 (53.1)
9d	Have the interactions you've had with staff during your mealtimes been positive?	6 (7.4)	0 (0.0)	1 (1.2)	1 (1.2)	9 (11.1)	64 (79)
	**Food ordering system**						
10b	Are the meals that you order from the menu the meals that you receive?	11 (13.6)	1 (1.2)	2 (2.5)	8 (9.9)	14 (17.3)	45 (55.6)
	Are the main meals served at an appropriate time for you?	2 (2.5)	0 (0.0)	3 (3.7)	4 (4.9)	14 (17.3)	58 (71.6)
	Are the staff who bring and take your menu friendly and polite?	8 (9.9)	0 (0.0)	1 (1.2)	0 (0.0)	4 (4.9)	68 (84)

The physical environment, the food ordering system and staff interactions and assistance were consistently rated higher than the food quality domain. Most participants (> 70%) reported that the physical environment, including noise (5a); odours (5e); visitors and other patients (5b); and room surroundings (5c) ‘rarely’ or ‘never‘ impacted their ability to eat. Staff interactions were highly rated, with over 90% of participants reporting interactions with staff (9 d) as ‘often’ or ‘always’ positive. Staff assistance and availability (9b and 9c) were the poorest‐performing indicators in the staff interactions dimension. A vast majority of participants rated meal timing and friendliness of menu monitors (10d) as ‘often’ or ‘always’ positive (89% and 89% respectively), whilst 73% of patients rated meal accuracy (10c) as ‘often’ or ‘always’ occurring.

## 5 Qualitative Findings

**Figure 1:**
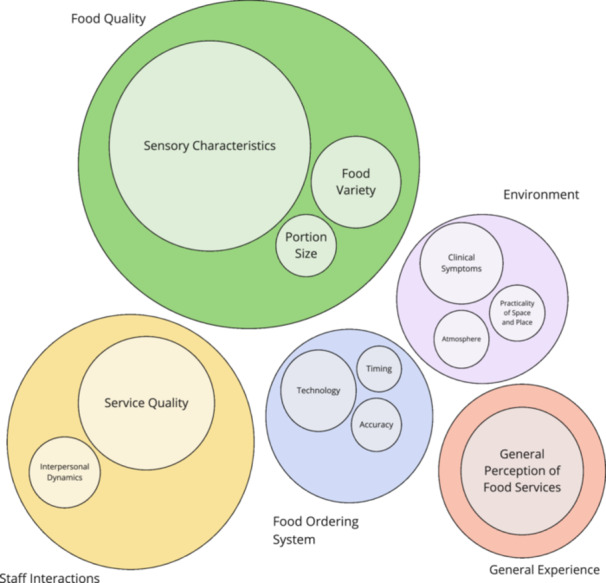
Bubble chart representing the thematic analysis from the modified AMPHET. Major themes are represented by the large circles and subthemes are represented by the smaller embedded circles. The size of the bubbles corresponds to the relative frequency of which themes and subthemes were coded in the thematic analysis.

Four key themes aligning with the four dimensions of MTE, and eleven subthemes emerged from the deductive thematic analysis (Figure 1, Table 3). A fifth theme, *General Experience* and the subtheme *General Perception of Food Service* was identified throughout all MTE dimensions, which encapsulated broad patient opinions on food service experience, general assessments of food quality and food service logistics.

**Table 3:** Thematic analysis.

Theme	Subtheme	Coding frequency (*n*)	Subtheme definition	Coding frequency: Theme perceived positively	Exemplar quote(s)	Coding frequency: Theme perceived negatively	Exemplar quote(s)
Food Quality	Sensory characteristics	69	Refers to the qualities of food that are perceived by taste, aroma, texture, temperature and appearance to influence how individuals interpret, experience and evaluate the acceptability of food.	6	‘The main meals have good flavour’ (female, 32, LOS* 1 day)	63	‘Sometimes the food is too cold. It is usually more peppery or spicey and needs a lot more salt. The pastry for a pie I once had was mushy and not crunchy and the beef is too tough with no flavour’ (female, 89, LOS* 3 days) ‘There are a couple of items I wouldn't have picked. A couple of times there's a meal that doesn't look good. The casserole/stew is all one colour. It looks all the same’ (male, 57, LOS* 6 days)
	Food variety	22	Encompasses the range and diversity of food choices available to meet individual food preferences, dietary and cultural needs. This theme also considers the repetition of meals and foods within the menu cycle.	3	‘Some of the meals you get quite a few choices. Everything is fine’ (male, 56, LOS* 2 days)	19	‘They need to be more generous with fruit and vegetables and things in season. They need more options. I reckon the foods have been the same at every meal’ (female, 68, LOS* 6 days) ‘It is repetitive. No variety at all. I have memorised the menu. Food is always horrible’ (female, 61, LOS* 75 days)
	Portion size	8	Perceived appropriateness of meal portion sizes in relation to appetite and preferences.	1	‘Really satisfied. I ordered risotto and it was a great size.’ (female, 70, LOS* 5 days)	7	‘As my appetite returned there was too much on the plate for me’ (male, 83, LOS* 14 day) ‘The kitchen hasn't been able to accommodate for smaller sized meals’ (female, 71, LOS* 12 days)
Environment	Atmosphere	6	Encompasses how various elements of the environment, including noise levels, lighting, odours, presence of visitors or patients and or/interruptions by staff members may impact overall mealtime experience.	3	‘The environment is pretty good’ (female, 74, LOS* 1 day)	3	‘I find in shared rooms the other person's television can be too loud. I prefer being in open spaces and don't like having the curtains separate the two patients in the room’ (female, 89, LOS *3 days)
	Practicality of place and space	7	Encompasses the practical aspects of the mealtime setting, including the accessibility of the meal tray, and layout and furniture of the patient room.	1	‘There is always room for the tray to sit on the table’ (female, 79, LOS* 5 days)	6	‘I am bedbound, if the tray is not close it just sits there’ (female, 36, LOS* 9 days)
	Clinical symptoms	19	Focuses on how various symptoms, such as loss of appetite, nausea, vomiting, pain, tiredness, difficulty chewing or swallowing and/or mood may impact food intake during mealtimes.	0		19	‘If I am fasting on and off like in the last few days or if I am feeling unwell or in pain it is a factor that can reduce the amount I can eat’ (male, 74, LOS* 9 days) ‘Feeling very tired and nauseous, just not feeling well.’ (female, 42, LOS* 1 day)
Staff Interactions/Assistance	Service Quality	33	Encompasses behaviours, actions and perceived professionalism of staff during mealtimes. Includes aspects such as timeliness, attentiveness, adherence to protocols and how staff handle meal ordering, interruptions, meal delivery, or assistance during eating.	17	‘The staff wake me up when the meal arrives which is good to know I have the choice whether to wake up and eat or go back to sleep’ (female, 49, LOS* 8 days) ‘Although it's not the food service staffs role to fill up the water, one of the food service ladies did when my water was low which was very nice’ (female, 70, LOS* 9 days)	17	‘After 3 days, the menu monitors stop coming in my room and I get a horrible default meal’ (female, 61, LOS* 75 days) ‘Sometimes the packaging is difficult. Our meals coincide with staff mealtimes. You'll be lucky if you find someone’ (female, 73, LOS* 4 days)
	Interpersonal dynamics	16	Focuses on interpersonal communication and personal interactions with staff, including perceived attitudes, display of empathy and friendliness, and non‐verbal cues such as body language.	13	‘The staff are very friendly and cooperative and helpful’ (male, 74, LOS* 9 days) ‘The Kitchen staff have been lovely’ (female, 68, LOS* 2 days)	3	‘I have had not so nice interactions with kitchen staff. I didn't know that there was an option to type in a request and I felt like the food service staff weren't overly nice when I was trying to explain the confusion’ (female, 40, LOS* 25 days)
Food ordering system	Timing	10	Addresses suitability of mealtime scheduling, the intervals between meals, the timing of meal orders and the promptness of meal delivery.	0		10	‘Tea is served at 4:30 pm it is ridiculous. I usually have tea at 7:00 pm’ (female, 72, LOS* 1 day)
	Accuracy	11	Focuses on how accurately the meals delivered match the items ordered from the menu.	2	‘90% of the meals are correct’ (male, 75, LOS* 4 days)	9	‘Delivery for the wrong patient with the nutrition supplement. I did not get a spoon with my porridge and did not get sugar with it.’ (female, 61, LOS* 75 days)
	Technology	15	Encompasses efficiency and functionality of the electronic meal ordering (EMO) system, including how well it allows patients to select meals and whether it supports accuracy and ease of use. It considers factors such as user‐friendliness and reliability.	5	‘The electronic system is easy to use’ (female, 32, LOS* 1 day)	10	‘At times it can be clunky. Once, someone had to come and check it. Sometimes the screen doesn't work. Where, you hit “order now” the icons are really small. You have to almost use your pinky nail. It's a fine area to hit.’ (male, 57, LOS* 6 days) ‘The PES is not easy to use. Using your finger, it's very easy to miss something. The screen doesn't work.’(female, 67, LOS* 4 days)
General Experience	General Perception of Food Service	37	Reflects patients' overall satisfaction with meal service, including general assessment of food quality, meal logistics and broad opinions on the food service experience.	22	‘I think logistics for hospital in the timeframe they do a good job. Overall, meals are high standard given the logistics’ (male, 57, LOS* 6 days)	15	‘I completed a feedback survey last year and since coming back have only seen one improvement ‐ which is an improvement in them mashed potatoes however the rest is all the same. As I have a pancreatic condition, it is important I try to keep eating but I don't want to eat the food so I have had to outsource it which has somewhat increased my anxiety. I feel that the reason it hasn't changed is because of funding.’ (female, 60, LOS* 4 days)

### 5.1 Food Quality: Sensory Characteristics, Food Variety and Portion Sizes

Comments related to the food quality domain dominated the results. Patients were most frequently dissatisfied with the sensory characteristics of meals. Comments relating to meals being unappealing in flavour, texture and appearance were common. Patients commonly commented on the lack of diverse meal options and the repetition of dishes in the menu cycle. Suggestions for improvement often highlighted the need for increased variety in meal choice, the use of fresher ingredients and variation in cooking methods. A small number of patients indicated that portion sizes were too large, particularly among those with reduced appetite.

### 5.2 Environment: Atmosphere, Practicality of Space and Place and Clinical Symptoms

Comments on clinical symptoms were most prevalent in the responses. Patients highlighted that symptoms such as low appetite, pain and fatigue were impacting mealtimes most frequently. A small number of patients cited disturbances such as loud noises, lack of privacy and accessibility of meal trays and room layout as being barriers to mealtime enjoyment.

### 5.3 Staff Interactions and Assistance: Staff Service Quality and Interpersonal Dynamics

Service quality dominated comments surrounding staff interactions. Mixed results were observed. Some patients highlighted staff being ‘helpful’ and ‘professional’, whilst others reported issues with receiving adequate assistance and/or timely care during mealtimes. These issues were commonly attributed to staff being ‘too busy’ or having ‘no time’. Nevertheless, interpersonal dynamics between staff and patients were viewed largely positively. Patients perceived staff to be ‘friendly’ and ‘cooperative’.

### 5.4 Ordering System: Meal Timing, Accuracy and Technology

Technology was the most frequently coded subtheme that arose from the ordering system dimension. Patients commonly reported difficulties with the PES, particularly its functionality and interface design. Issues such as unresponsive or malfunctioning screens and confusing design were commonly discussed. Several patients found mealtimes too early or felt that gaps between meals were too short. A small number of patients noted issues with meal accuracy, such as receiving the wrong meal, delivery of oral nutrition supplementation to the wrong patient, or missing items on meal trays.

### 5.5 General Experience: General Perception of Food Services

General perception of food services emerged as a key subtheme, with comments encapsulating patients' overall satisfaction with food services and reflecting broader opinions that factored in experiences with logistics, comparison of food services with other health services and overall impact on well‐being. Comments were mixed, with some patients praising logistical efforts and high standard of meals, whilst other expressed general dissatisfaction with food services, including minimal improvements over time and low quality of food compared to other health services.

## 6 Discussion

This cross‐sectional study of MTE was conducted at Bendigo Health using a modified version of the AHPMET tool integrated quantitative data with qualitative feedback, enabling the exploration of the underlying contextual factors that influence MTE in an acute care population. Satisfaction with food quality – particularly in flavour, texture, appearance and variety was the least favourably rated aspect of the MTE in both the quantitative and qualitative results. Staff interactions were found to be overwhelmingly positive in this study, although staff availability and assistance in the context of mealtime care were found to be barrier to MTE in both the quantitative and qualitative responses.

Whilst the quantitative results revealed that approximately half to three quarters of participants reported that nutrition impact symptoms (NIS) rarely or never impacted intake, NIS were prominently discussed in the qualitative responses. This contrast in data highlights the limitations of relying exclusively on quantitative data to assess patient experience. In this study, qualitative feedback offered contextual data that was not fully represented in the quantitative results, reinforcing the value of capturing subjective patient experiences to meaningfully inform service improvement. The physical environment and meal ordering system were generally rated favourably; however, there was a relatively low uptake of the EMO system, and usability issues were highlighted in the qualitative responses.

This study found that food quality was the poorest‐performing dimension of MTE. Patients were most frequently dissatisfied with the sensory characteristics and variety of meals on offer. Dissatisfaction with food quality in hospital settings has been widely reported in the literature [13, 19, 31, 32, 33, 34]. Research has consistently demonstrated that overall patient satisfaction with hospital food services is greatly influenced by factors such as meal variety, flavour, texture and visual appeal [13, 19, 31, 32, 33, 34]. Whilst interventions aimed at enhancing food quality—such as improving flavour [35, 36], visual appeal [37, 38] and expanding menu choice [39, 40, 41, 42] —have shown positive outcomes in increasing patient satisfaction and dietary intake, there is limited research specifically evaluating interventions aimed at improving the overall sensory aspects of hospital meals, such as taste, texture and aroma [12].

Nutrition impact symptoms (NIS) including pain, nausea and fatigue were reported to be barriers to intake in this study. A recent cross‐sectional study in an acute population found that 81% of nutritionally at‐risk patients experienced NIS after four or more days of admission [43]. Additionally, a retrospective study across five Australian hospitals found that 89% of patients clinically assessed to have hospital‐acquired malnutrition experience NIS [9]. Given the strong association between NIS, reduced intake and poorer clinical outcomes [2, 9, 44], targeted interventions in the management of NIS become critical. Despite this, NIS are underrecognised and undertreated in the hospital setting [45]. Pharmacological interventions including the use of antiemetics and effective pain management play an important role in mitigating NIS [39, 46]. Likewise, as specialised nutrition professionals, dietitians play a key role in providing individualised nutrition care and personalised dietary modification in addressing both the physical and psychosocial factors impacting NIS and intake [43]. Thus, timely and regular screening for NIS is crucial in ensuring early identification and interventions to better address patients' nutritional needs [43]. This underscores the importance of a multidisciplinary approach to identifying NIS, including early dietetic support in managing NIS to improve nutritional intake and ultimately in preventing malnutrition [43, 47].

Consistent with previous food service satisfaction studies, interpersonal dynamics between staff and patients were perceived to be highly positive [12, 19, 34]. However, inadequate mealtime assistance was also highlighted in this study. In the hospital setting, responsibility for mealtime assistance typically fall on nursing staff [48, 49]. Workload, competing priorities and time pressures are widely recognised as barriers to meeting patient mealtime needs in acute care settings [50, 51, 52, 53, 54]. Mealtime assistance, including adequately positioning patients, timely assistance and adequate tray set‐up are known contributors to improved intake and overall patient satisfaction [14, 19, 48]. Multidisciplinary solutions that integrate a range of hospital staff – including Dietitian Assistants, other Allied Health Assistants and Food Service Officers into mealtime care have been suggested as strategies to improve mealtime support [50]. A recent systematic review found that delegation of several components of the nutrition care process – including mealtime assistance to Dietitian Assistants improved nutritional intake and patient outcomes, including decreased mortality and length of hospital stay [55]. Such strategies have the potential to provide more consistent mealtime care to patients whilst alleviating the burden on nursing staff.

This study found a relatively low uptake of the EMO system among patients. Technical difficulties with touchscreen technology and confusion with the interface were commonly reported. These findings align with previous research demonstrating that patients' engagement with, and acceptance of EMO are strongly shaped by perceived usability and interface design [56]. Difficulties in navigating systems and in using touchscreen technology also appear to be influenced by age, with older adults reporting greater challenges in the literature [57]. It is particularly important to take age‐related differences in accessing hospital technologies into account, given the growing proportion of older adults accessing inpatient care [58]. While interventions such as tailored education and ongoing IT support have shown some success in enhancing self‐efficacy and uptake of hospital technologies [56, 59, 60], emerging evidence identifies user‐centred design as a critical enabler to effective implementation and engagement [60, 61]. Involving patients directly in the development of technological solutions in EMO through user testing and feedback has been associated with fewer interface‐related challenges and improved usability [61]. These findings reinforce the importance of genuine end‐user involvement in ensuring EMO systems are accessible and acceptable among patient populations with varying levels of digital literacy.

## 7 Strengths and Limitations

A major strength of this study is that it was the first to apply the AHPMET to measure MTE in an acute inpatient population. The AHPMET captured the breadth and depth of factors influencing MTE, including qualitative patient perspectives not previously addressed by existing tools in acute care settings. This triangulation of data can inform targeted strategies in quality improvement in food services and mealtime care practices. The use of an interviewer‐administered tool removed the requirement for patient literacy and therefore broadened the generalisability of study results.

It is important to acknowledge that this study had several limitations. This study was administered at a single, regional hospital site, limiting the generalisability and external validity of the findings. The exclusion of patients who were too unwell to participate (including those in ICU), non‐English speaking patients and those with significant cognitive impairment may have omitted a cohort of patients who are at high nutritional risk. Additionally, the relatively small sample size may limit the generalisability of the findings. As the survey was interviewer‐administered, there is the potential for both interviewer and social desirability biases. However, as researchers who administered the surveys were independent, third‐party, nonfood service‐related staff, this may have helped mitigate this. Additionally, as a standardised script was used for survey administration, this may have enhanced neutrality, reducing the likelihood of interviewer influence.

## 8 Future Directions

To improve the robustness of this study's results, future studies are needed to test the AHPMET in a larger sample size and among patients who were not well represented in this sample, including patients with non‐English speaking backgrounds, patients with significant cognitive impairment and patients deemed to be too unwell to participate (including those in ICU) in data collection. To ensure equitable access to providing feedback, multiple methods of survey administration may be considered, including self‐administration via electronic or printed surveys, the use of interpreters and/or through family or carers acting as proxies when needed [62, 63]. Providing the option for self‐administration may mitigate the risk of social desirability bias and enhance the rigour of future studies [64].

This study provides robust data that may be used to justify targeted investment in food service improvement. The dissemination of this study's findings to key stakeholders including hospital leadership and clinical teams may facilitate the prioritisation of food services, foster multidisciplinary collaboration and support the integration of patient feedback into future policy [65]. By highlighting the direct impact of food quality on nutritional intake and patient experience, this study places an emphasis on food services as a critical component of person‐centred care and improved patient outcomes. In addition to prioritising improvements to food quality, health services should prioritise user‐centred design of EMO systems [56], given the potential for EMO in improving patient satisfaction and dietary intake [59, 66]. Consultation with patients through codesign, usability testing and iterative feedback has been shown to be both feasible and effective in hospital settings [61].

## 9 Conclusion

This study captured a range of factors that impact MTE among patients in a regional hospital setting. Addressing the multifactorial dimensions that impact MTE is complex, and integrating patient perspectives and care values into service planning is essential for fostering a more patient‐centred hospital foodservice experience. Ongoing monitoring of MTE is vital to ensure that mealtime care and food service operations are evidence‐based and patient‐centred. The AHPMET tool addresses the breadth of factors associated with MTE whilst allowing for qualitative feedback, offering a nuanced understanding of MTE, which is essential for driving quality improvement.

## Author Contributions

Conceptualisation: Kate Furness, Gina Trakman, Helen Ussher and Virginia Fox. Methodology: Kate Furness, Gina Trakman, Helen Ussher, Lauren Ballantyne. Student supervision during data curation: Helen Ussher. Data curation: Laura Lam, Amy Daglas and Ella Hamilton. Quantitative data analysis: Laura Lam, with supervision from Kate Furness and Gina Trakman. Qualitative thematic analysis: Laura Lam and Amy Daglas with supervision from Kate Furness. Original draft manuscript preparation: Laura Lam with review and editing from Kate Furness and Gina Trakman. Writing, review and editing: all authors. All authors have read and agreed to the published version of the manuscript.

## Ethics Statement

This study met the Bendigo Health Quality Assurance threshold and was exempted from ethics approval.

## Consent

Informed verbal consent was obtained from all subjects involved in this study.

## Conflicts of Interest

Virginia Fox, Lauren Ballantyn and Helen Ussher hold leadership positions at the Health Service that was the site of recruitment, but were not involved with direct participant recruitment, obtaining informed consent, data collection, or data analysis in this study.

### Peer Review

The peer review history for this article is available at https://www.webofscience.com/api/gateway/wos/peer-review/10.1111/jhn.70068.

## Supporting information

File S1: Modified AHPMET survey.

File S2: Standardised pre‐interview script.

## Data Availability

The data that support the findings of this study are available from the corresponding author upon reasonable request. The data presented in this study are available upon reasonable request from the corresponding author.
