# Supplementary material for: Exploring Patient Mealtime Experience in an Acute Care Setting Using the Modified Austin Health Patient Mealtime Experience Tool
Source: J Hum Nutr Diet. 2025 May 28;38(3):e70068. doi: 10.1111/jhn.70068 (PMC12120381; doi:10.1111/jhn.70068)
Supplement: Supplementary file 1 — File S1: Modified AHPMET survey. [file JHN-38-0-s001.pdf]

Study Code:
Date Completed:

Demographics (Patient Reported Data)

How old are you (in years)? ______ Prefer not to say
What is your gender?
Male / Female / Transgender Male / Transgender Female / Non-Binary / Prefer not to say
What country were you born in?
Australia Another country (please specify _____________) Prefer not to say
What language do you speak at home?
English Another language (please specify _____________) Prefer not to say
Do you have a disability?
Yes No Prefer not to say
Do you identify as Aboriginal or Torres Strait Islander?
Yes No Prefer not to say

Contextual Data (Data Collected by Interviewer)

Ward

____

Treating Team

____

Current diet codes

____

Length of stay

____

Patient Mealtime Experiences at Bendigo Health (Patient Reported Data)

This survey allows you the opportunity to share your opinion and experience of mealtimes during your stay at Bendigo Health. Your responses will help us to make improvements to mealtimes in the Bendigo Hospital.

This survey will ask questions what affects your enjoyment of meals while you are in hospital. The survey will include questions about:

- The environment (i.e. noise, comfort) in which you eat your meals
- The quality of food (including taste, presentation, temperature and variety)
- Interactions with staff and/or visitors
- Assistance provided during your mealtimes
- Food ordering system

1. **How many days have you been in hospital for?**

   ____

2. **Do you know what your current diet code(s) are? If yes, can you tell us what the diet code(s) are?**

____

3. **Please rate how you're feeling today:**

Low ☆ ☆ ☆ ☆ ☆ Great

a) **Why?**

____

Food Quality

4. **For each of the following questions, please select one answer.**

a) **How frequently have you been satisfied with the quality of the food you have received at Bendigo Health?**

Always Often Sometimes Rarely Never Not Applicable

b) **Have the meals offered been appropriate for your beliefs or needs? (e.g. religious, cultural, vegan)?**

Always Often Sometimes Rarely Never Not Applicable

c) **Has there been variety in your meal choices?**

Always Often Sometimes Rarely Never Not Applicable

d) **Has the serving size of your meals been adequate?**

Always Often Sometimes Rarely Never Not Applicable

e) **Have the meals been served at a suitable temperature?**

Always Often Sometimes Rarely Never Not Applicable

f) **Have the meals looked appetising when they were presented?**

Always Often Sometimes Rarely Never Not Applicable

g) **Has the taste and flavour of the meals been to your liking?**

Always Often Sometimes Rarely Never Not Applicable

h) **Overall, have your meals been enjoyable?**

Always Often Sometimes Rarely Never Not Applicable

i) **Why/Why not?**

____

____

____

Environment

The following questions relate to the environment/space/room in which you sit and eat your meals. For each of the following questions, please circle one answer.

5. **Do the following factors affect the amount of food you eat during mealtimes?**

a) **Noise**

Always Often Sometimes Rarely Never Not Applicable

b) **Visitors and/or other patients**

Always Often Sometimes Rarely Never Not Applicable

c) **Room surroundings (e.g. layout of the room, furniture, lighting, ambience)**

Always Often Sometimes Rarely Never Not Applicable

d) **Interruptions by hospital staff (e.g. wanting to speak to you or give you treatment)**

Always Often Sometimes Rarely Never Not Applicable

e) Smells and odours

Always Often Sometimes Rarely Never Not Applicable

6. Is your meal tray placed where you can easily reach your food?

Always Often Sometimes Rarely Never Not Applicable

7. Is your tray table cleared of clutter (medical products, specimen samples, urinals) when your meal is served?

Always Often Sometimes Rarely Never Not Applicable

Please provide any further comments regarding your mealtime environment:

____

____

____

8. Over the past 2 days have the following aspects affect the amount of food you eat during mealtimes?

a) Loss of appetite

Always Often Sometimes Rarely Never Not Applicable

b) Nausea and/or vomiting

Always Often Sometimes Rarely Never Not Applicable

c) Pain

Always Often Sometimes Rarely Never Not Applicable

d) Tiredness

Always Often Sometimes Rarely Never Not Applicable

e) Difficulty chewing or swallowing

Always Often Sometimes Rarely Never Not Applicable

f) Position (e.g. your posture, ease of access to food tray)

Always Often Sometimes Rarely Never Not Applicable

Please specify any other factors affecting the amount of food you eat during mealtimes?

____

____

____

____

Staff Interactions/Assistance

9. For each of the following questions, please select one answer.

a) Does the meal tray (including cutlery, serviettes, packaging etc.) have everything you need?

Always Often Sometimes Rarely Never Not Applicable

b) Is assistance available if you need help opening the packaging on the meal tray?

Always Often Sometimes Rarely Never Not Applicable

c) When you need help, are staff there to provide assistance at your mealtimes?

Always Often Sometimes Rarely Never Not Applicable

d) Have the interactions you've had with staff during your mealtimes been positive?

Always Often Sometimes Rarely Never Not Applicable

Please provide any further comments about your staff interactions/assistance:

Food Ordering System

10. For each of the following questions, please select one answer.

a) How do you order food from the menu?

- ❑ Patient Entertainment System (your bedside screen)
- ❑ Menu monitor
- ❑ Family / visitors order for me
- ❑ I don't order my meals
- ❑ Other (free text comment)

____

b) Are the meals that you order from the menu the meals that you receive?

Always Often Sometimes Rarely Never Not Applicable

c) Are the main meals served at an appropriate time for you?

Always Often Sometimes Rarely Never Not Applicable

d) Are the staff who bring and take your menu friendly and polite?

Always Often Sometimes Rarely Never Not Applicable

Please provide any further comments about ordering your meals:

____

____

11. Short Answer

a) Please provide any further comments about your mealtime experience at Bendigo Health:

____

____

____

____

b) Did you require assistance from staff with completing this survey?

- ❑ Yes
- ❑ No

c) Further comments regarding the quality of food:

____

____

____

____
