# Supplementary material for: Exploring Patient Mealtime Experience in an Acute Care Setting Using the Modified Austin Health Patient Mealtime Experience Tool
Source: J Hum Nutr Diet. 2025 May 28;38(3):e70068. doi: 10.1111/jhn.70068 (PMC12120381; doi:10.1111/jhn.70068)
Supplement: Supplementary file 2 — File S2: Standardised pre‐interview script. [file JHN-38-0-s002.docx]

You are invited to take part in this research project, which is called ‘Patient Mealtime Experiences at Bendigo Health’.

Bendigo Health is interested in patients mealtime experiences. This is because a patient’s experience of mealtime services can impact their nutritional status.

Bendigo Health is conducting this research to implement strategies that can help improve the mealtime experience based on patient feedback

This is a stand-alone project and the results will be used as part of our Research Project at La Trobe University.

Participation in this project is **voluntary**. If you do not wish to take part, you do not have to. It will not affect the healthcare you receive from Bendigo Health.

If you decide to participate, you will complete a 10-20 minute interviewer-administered survey about your mealtime experience at Bendigo Health. During the interview, you will be required to answer questions about what affects your enjoyment of meals while you are in hospital.
